# Deep learning based sarcopenia prediction from shear-wave ultrasonographic elastography and gray scale ultrasonography of rectus femoris muscle

**DOI:** 10.1038/s41598-022-07683-6

**Published:** 2022-03-04

**Authors:** Jisook Yi, YiRang Shin, Seok Hahn, Young Han Lee

**Affiliations:** 1 Department of Radiology, Haeundae Paik Hospital, Inje University College of Medicine, Busan, South Korea; 2 Department of Radiology, Research Institute of Radiological Science, and Center for Clinical Imaging Data Science (CCIDS), Yonsei University College of Medicine, 50-1 Yonsei-ro, Seodaemun-gu, Seoul, 03722 South Korea

**Keywords:** Biomarkers, Medical research

## Abstract

We aim to evaluate the performance of a deep convolutional neural network (DCNN) in predicting the presence or absence of sarcopenia using shear-wave elastography (SWE) and gray-scale ultrasonography (GSU) of rectus femoris muscle as an imaging biomarker. This retrospective study included 160 pair sets of GSU and SWE images (n = 160) from December 2018 and July 2019. Two radiologists scored the echogenicity of muscle on GSU (4-point score). Among them, 141 patients underwent CT and their L3 skeletal muscle index (SMI) were measured to categorize the presence or absence of sarcopenia. For DCNN, we used three CNN architectures (VGG19, ResNet-50, DenseNet 121). The accuracies of DCNNs for sarcopenia classification were 70.0–80.0% (based on SWE) and 65.0–75.0% (based on GSU). The DCNN application to SWE images highlights the utility of deep-learning base SWE for sarcopenia prediction. DCNN application to SWE images might be a potentially useful biomarker to predict sarcopenic status.

## Introduction

The term “sarcopenia” was coined in 1988 by Rosenberg and was originally defined as a muscle loss in the appendicular muscle mass in older people^1^. In 2010, the definition was modified to also refer to low muscle function^2^. Muscle strength differs among individuals and decreases with age^3^. Muscle mass measurement for sarcopenia evaluation is usually assessed by medical imaging modalities, such as dual-energy X-ray absorptiometry, computed tomography (CT), magnetic resonance imaging (MRI), and ultrasonography (USG).

USG can measure targeted muscle size and echogenicity in real-time with several advantages (e.g., relatively low cost, portability, lack of radiation exposure, provision of muscle components such as fibrosis, adipose tissue infiltration). The thickness of the quadriceps is known to be highly correlated with voluntary contraction force, and the echogenicity of the rectus femoris muscle is known to be associated with muscle strength^4,5^. Intramuscular fat and fibrosis affect the echogenicity of gray-scale USG (GSU), and the qualitative information is essential for evaluating the functional information; it is not always correlated with quantitative information^6^. Shear-wave elastography (SWE) USG is a relatively new non-invasive functional imaging method for measuring soft tissue elasticity (i.e., tissue stiffness) with high reproducibility and objective quantitative imaging capability, which might contain more objective qualitative information^7^. In the evaluation of idiopathic inflammatory myopathy, the SWE measurements in Young’s modulus of the muscle demonstrated significant associations with disease activity, suggesting that it could be considered as a new modality for monitoring disease activity^8^.

Imaging analysis and interpretation of radiologic imaging are basic tasks performed by radiologists in providing qualitative radiologic reading, depending on their experience and medical knowledge. However, in this era of big data and artificial intelligence, radiologic imaging has been enhanced with a capability to provide quantitative imaging biomarkers for early detection, further characterization, activity monitoring, and response to treatment. Recently, deep learning and radiomics have been introduced in radiology for analyzing and interpreting images. Deep learning is a subset of machine learning where multiple hidden neural networks learn representations of data through simple and complex feature abstraction to perform tasks^9,10^. Convolutional neural networks (CNNs) are a type of artificial neural network designed to use pixel data in images to learn abstract representations with high levels of semantics, where deep convolutional neural network (DCNN) has been shown to exhibit high performance in medical classification, detection, and segmentation tasks [8–12]^9,11–14^. Radiomics is a method of high throughput data mining that extracts numerous image features from routine clinical images to assess tumor characteristics on radiologic images, such as spatial heterogeneity, texture, or shape for precision medicine and decision support^15,16^. However, to date, there has been no study that predicts the sarcopenia on muscle USG using either DCNNs or radiomics.

Therefore, the aim of this study is to evaluate the performance of a deep convolutional neural network (DCNN) in predicting the presence or absence of sarcopenia using shear-wave elastography (SWE) and gray-scale ultrasonography (GSU) of rectus femoris muscle as an imaging biomarker.

## Results

### Demographics

Among 160 subjects who underwent USG, 69.4% (111/160) were classified as low-grade muscle echogenicity (grade 0, n = 27; grade 1, n = 84), and the other 30.6% (49/160) were classified as high-grade muscle echogenicity (grade 2, n = 39; grade 3, n = 10). Among 141 subjects who underwent USG and CT, 20.6% (29/141) were categorized as “a sarcopenia” (men, n = 22; women, n = 7) and 79.4% (112/141) were categorized as “not a sarcopenia” (men, n = 34; women, n = 78%).

### Performance of the three DCNNs

**Figure 1:**
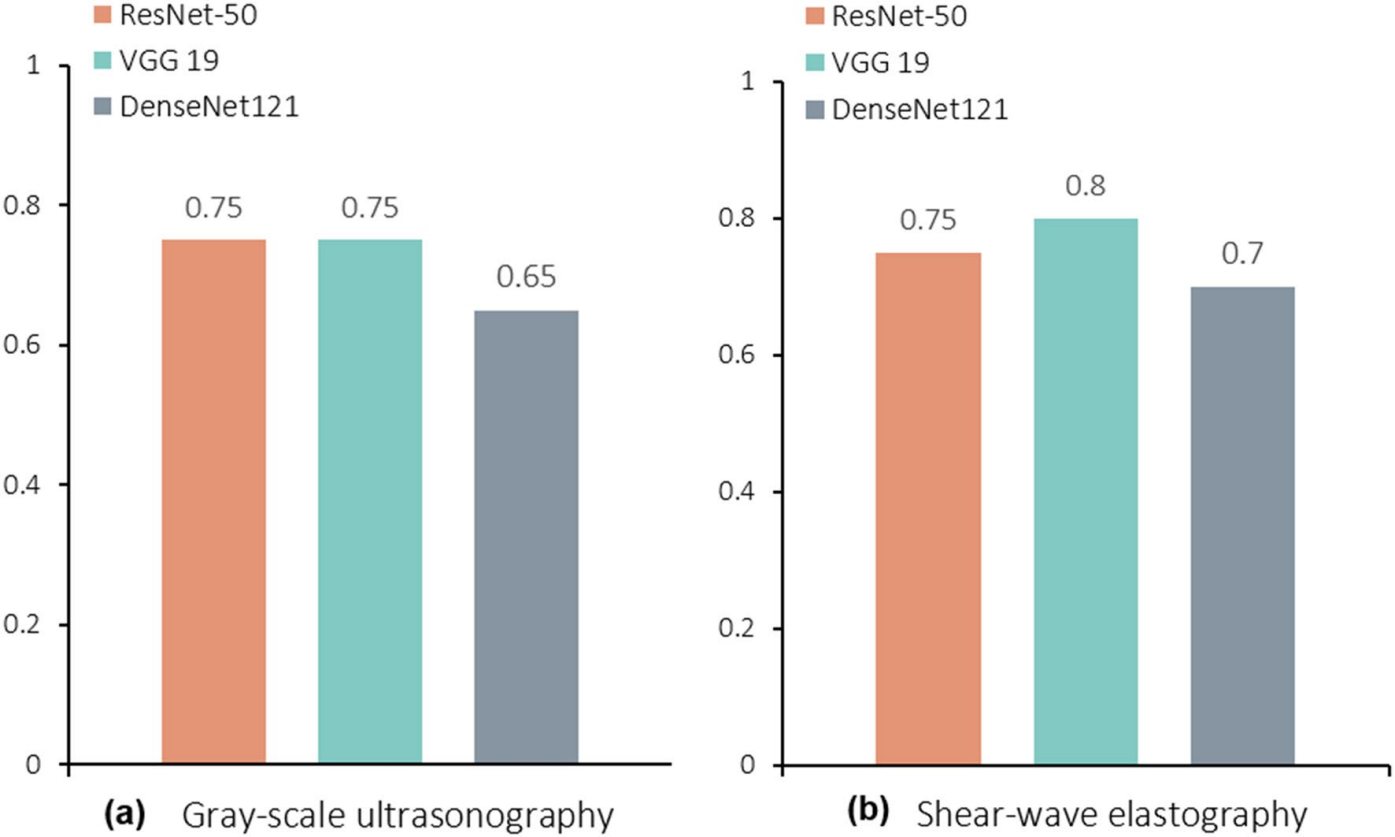
The accuracy of three deep learning neural network (DCNN) for predicting presence or absence of sarcopenia on (**a**) gray-scale ultrasonography and (**b**) shear-wave elastography.

**Figure 2:**
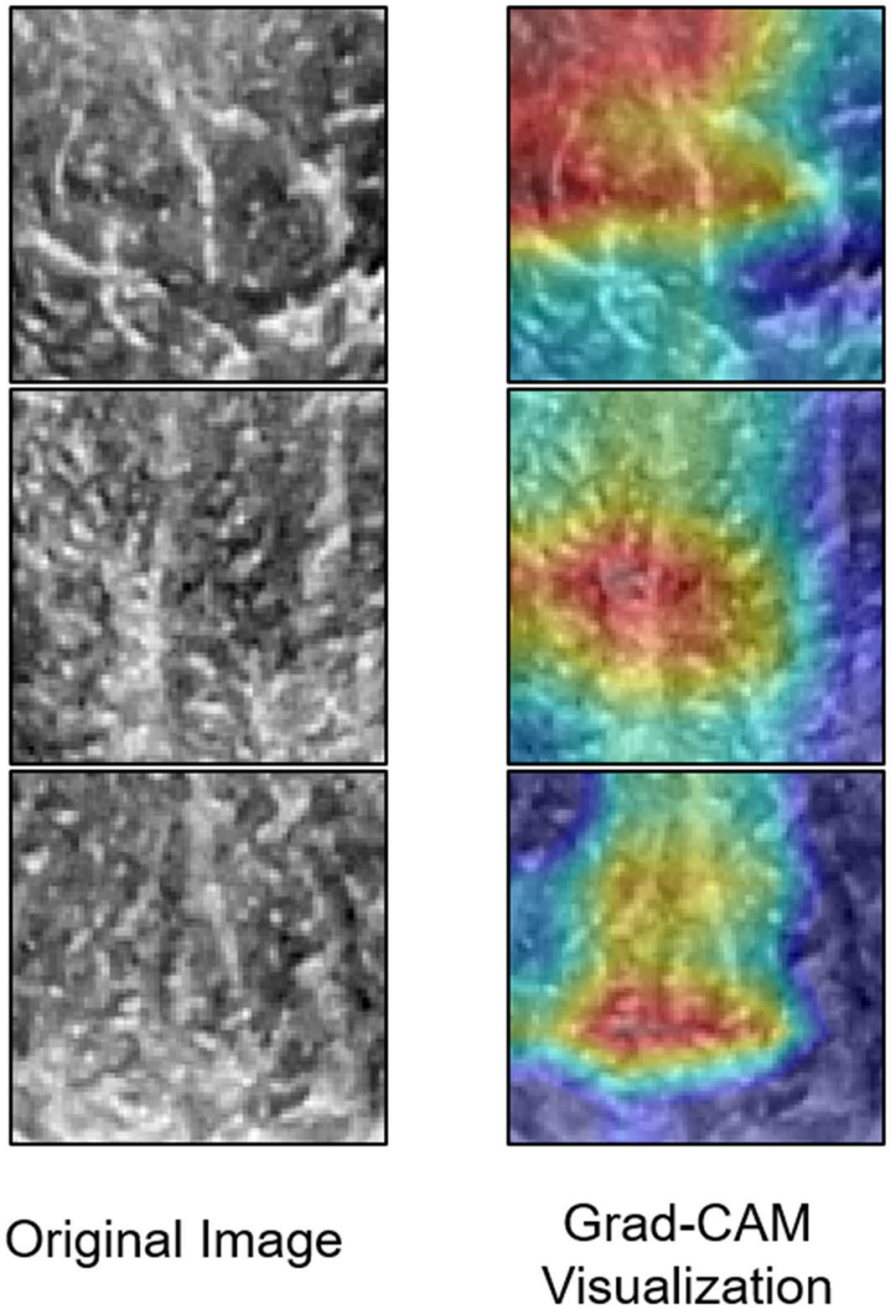
Example of DCNN model image input and Grad-CAM visualization of sarcopenia prediction architecture (VGG19). High activations are noted at hyperechoic muscle fascia/fibrosis or low echoic fat area.

The diagnostic performance of the three DCNNs in classifying the echogenicity of the muscle grades is shown in Table 1. The VGG19 and DenseNet121 pre-trained model outperformed ResNet model (accuracy, 85.0%) (Fig. 1). The best performing architecture (DenseNet121) showed a sensitivity of 83.3% and specificity of 85.7% for grading muscle echogenicity. The comparison of the diagnostic performance in sarcopenia classification on GSU and SWE USG by three DCNNs are shown in Table 1 and Fig. 1. Using data augmentation and image resizing, the accuracy of sarcopenia classification based on GSU images increased considerably from 65.0 to 75.0% on VGG19 pre-trained model, yielding performance of 77.8% sensitivity, 72.7% specificity, and 0.77 AUC. In predicting sarcopenia with GSU images, Grad-CAM was applied and showed high activations in hyperechoic areas due to muscle fascia/fibrosis and hypoechoic areas considered as intramuscular fat area (Fig. 2). The VGG19 pre-trained model with SWE USG images yielded the best performance of 80.0% accuracy, 88.9% sensitivity, and 72.7% specificity (Fig. 1b).

**Table 1:** Diagnostic performance of three DCNNs for sarcopenia status classification on GSU and SWE images.

	Prediction of sarcopenia					
	GSU			SWE		
	VGG19	ResNet50	DenseNet121	VGG19	ResNet50	DenseNet121
AUC	0.77	0.76	0.67	0.84	0.74	0.76
Sensitivity	77.80%	88.90%	66.70%	88.90%	77.80%	66.70%
Specificity	72.70%	63.60%	63.60%	72.70%	72.70%	72.70%
PPV	70.00%	66.70%	60.00%	72.70%	70.00%	66.70%
NPV	80.00%	87.50%	70.00%	88.90%	80.00%	72.70%
Accuracy	75.00%	75.00%	65.00%	80.00%	75.00%	70.00%

DCNN, deep learning convolutional neural network; GSU, gray-scale ultrasonography; SWE, shear-wave elastography; AUC, area under the receive operating curve; PPV, positive predicted value; NPV, negative predicted value.

## Discussion

Muscle mass is positively associated with muscle strength and is supported by epidemiologic cohorts^17,18^. However, some researchers found that muscle quality rather than quantity determines muscle function^6,19^. The definition for sarcopenia has been modified to include both muscle quantity and function. Skeletal muscle is composed of a group of muscle fibers, and muscle fiber is broadly classified into “slow twitch” (type I) and “fast switch” (type II)^20^. A selective reduced size and the atrophy of type 2 fiber are associated with sarcopenia^21^. Additionally, intramyocellular and intermuscular fat may increase with aging (myosteatosis)^22^. The suggested mechanism for these histologic alterations of aging muscles involves age-related change in activation, proliferation, and differentiation of quiescent skeletal muscle precursor “stem cells” into adipocytes.

Although MRI or CT is considered as gold-standard modality for evaluating the whole body’s composition, ultrasound is a simple, reliable, and non-invasive imaging modality for muscle imaging that provides muscle quality (i.e., histologic changes) and quantity (i.e., muscle mass or volumes) in a relatively short time without exposure to ionizing radiation. Investigators have reported inter-rater consistency in measuring the cross-sectional area of the muscle and echogenicity evaluation using USG, and it was less affected by the operator’s skill^23–25^. These results might support the role of USG as an easily assessable screening modality for evaluating sarcopenia.

In this current study, we applied DCNNs to classify the echogenicity of the muscle on GSU and to predict the presence of sarcopenia on both of GSU and SWE USG images. The results showed a good diagnostic performance (80.0% to 85.0% accuracy) in classifying muscle echogenicity on GSU images. This result is similar to a previous study that used DCNN to classify the liver cirrhosis on US images (85.7% accuracy), and it outperformed all five radiologists^13^. For predicting sarcopenia on both GSU and SWE USG, the DCNNs slightly performed better on SWE USG (70.0% to 80.0% accuracy) than GSU USG (65.0% to 75.0% accuracy). The prediction of sarcopenia on GSU showed an enhanced performance (65.0% to 75.0% accuracy) with an end-to-end DCNN approach compared to MLP using radiomic features. The significant feature attributes of VGG19 model predictions can be observed through Grad-CAM (Fig. 2).

We have shown that DCNN plays an important role in the proposed system in automatically extracting useful features from a limited dataset. DCNNs compared to radiomics-based networks avoid human hand-crafted feature extraction, which is time consuming and have inter-subject variations in image acquisition. Additionally, in the case of MLP with radiomic features, it is difficult to determine precisely how our model arrived at its decision due to the “black box” of the neural network^26^.Our results reveal that a combination of appropriate image pre-processing pipelines and pre-trained DCNN architecture selection serves as an automatic high-performing model for sarcopenia/muscle grade to overcome classification tasks with small datasets. Although MLP classifier combined with radiomic features results in a relatively low performance compared to end-to-end deep learning approach, they give insights into the possible approaches to improve performance and reproducibility of the learning systems to become fully quantitative imaging biomarkers.

There are some limitations to this study. First, the number of subjects was relatively small for deep learning, and the pre-trained networks were not directly optimized from ultrasound images. Nevertheless, this is the first study to determine the performance of DCNNs in evaluating muscle echogenicity grades and in diagnosing sarcopenia, yet it demonstrated a good performance. The performance is anticipated to improve with more suitable pre-trained network and a larger dataset. Second, we obtained data from a Korean cohort. Therefore, we used the definition of sarcopenia based on a one–population skeletal muscle index. Hence, the results may not be generalized to other groups. Further studies with various cohorts, including functional performance evaluation, are needed to evaluate the robustness of the network. Third, we did not evaluate the added value of SWE on conventional GSU image. The image features of GSU and SWE are complementary in a clinical setting. In current study, we cannot extract elastography images from fusion images of underlying GSU. As a future study, feeding the DCNN models with both GSU and SWE images to predict the presence or absence of sarcopenia is needed to image the accuracy of the model since the might provide complementary information.

In conclusion, DCNNs exhibited a high performance in sarcopenia in based on SWE images. The DCNN application to SWE images highlights the utility of deep-learning base SWE for sarcopenia prediction. DCNN application to SWE images might be a potentially useful biomarker to predict sarcopenic status.

## Methods

### Datasets

**Figure 3:**
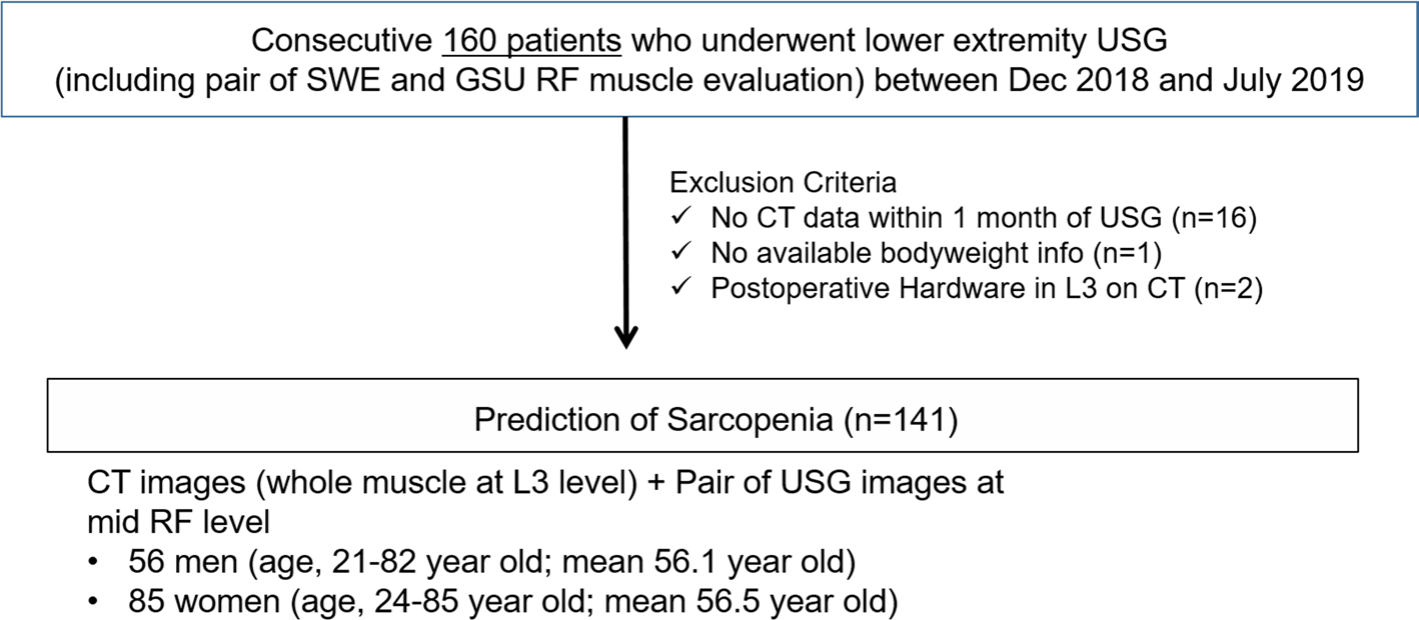
Flow diagram of study dataset.

This study was approved by the institutional review board of Inje University Haeundae Paik Hospital (Approval No. 2020-02-013). Informed consent was waived by the ethics committee/institutional review board of Inje University Haeundae Paik Hospital due to the retrospective design of the study. All research was carried out in accordance with relevant guidelines and regulations. Between December 2018 and July 2019, a total of 160 consecutive adult patients underwent lower extremity ultrasonography including GSU and SWE evaluation of the mid-third portion of the right rectus femoris muscle. The patients were included in the muscle echogenicity grading evaluation, and they comprised 62 men (aged 21–87 years; mean age, 57.4 years) and 98 women (aged 24–85 years; mean age, 56.4 years) (Fig. 3). For the muscle grade and sarcopenia prediction, we randomly selected 17 cases as the validation dataset for model selection and hyperparameter tuning, and 20 cases as test dataset to evaluate the performance of the model. For the prediction of sarcopenia, 19 subjects were excluded for the following reasons: (1) unavailable CT date within 1 month of USG (n = 16), (2) unavailable body weight information (n = 1), and (3) surgical hardware in L3 level on CT image (n = 2). Finally, 141 patients enrolled for sarcopenia classification, and it comprised 56 men (aged 21–82 years; mean age, 56.1 years) and 85 women (aged 24–85 years; mean age, 56.5 years). From the 141 cases, we randomly selected 17, 20, and 104 cases as the validation, test, and training sets, respectively. Collectively, the data sets were divided into training set, validation set, and test set (training:validation:test = 115:15:30 for muscle echogenicity and training:validation: test = 96:15:30 for sarcopenia prediction) (Fig. 3).

### Imaging study and analysis

#### Ultrasonography for muscle echogenicity grading and shear-wave elastography

**Figure 4:**
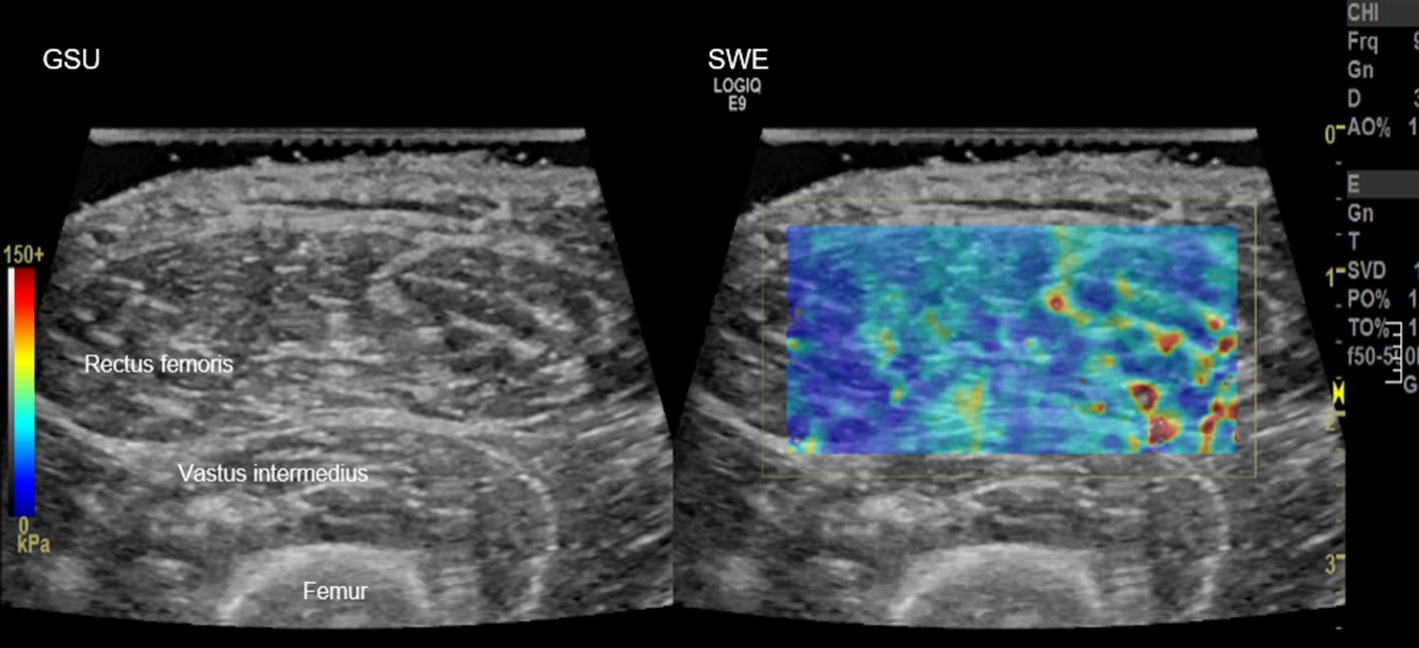
Representative image of ultrasonography.

**Figure 5:**
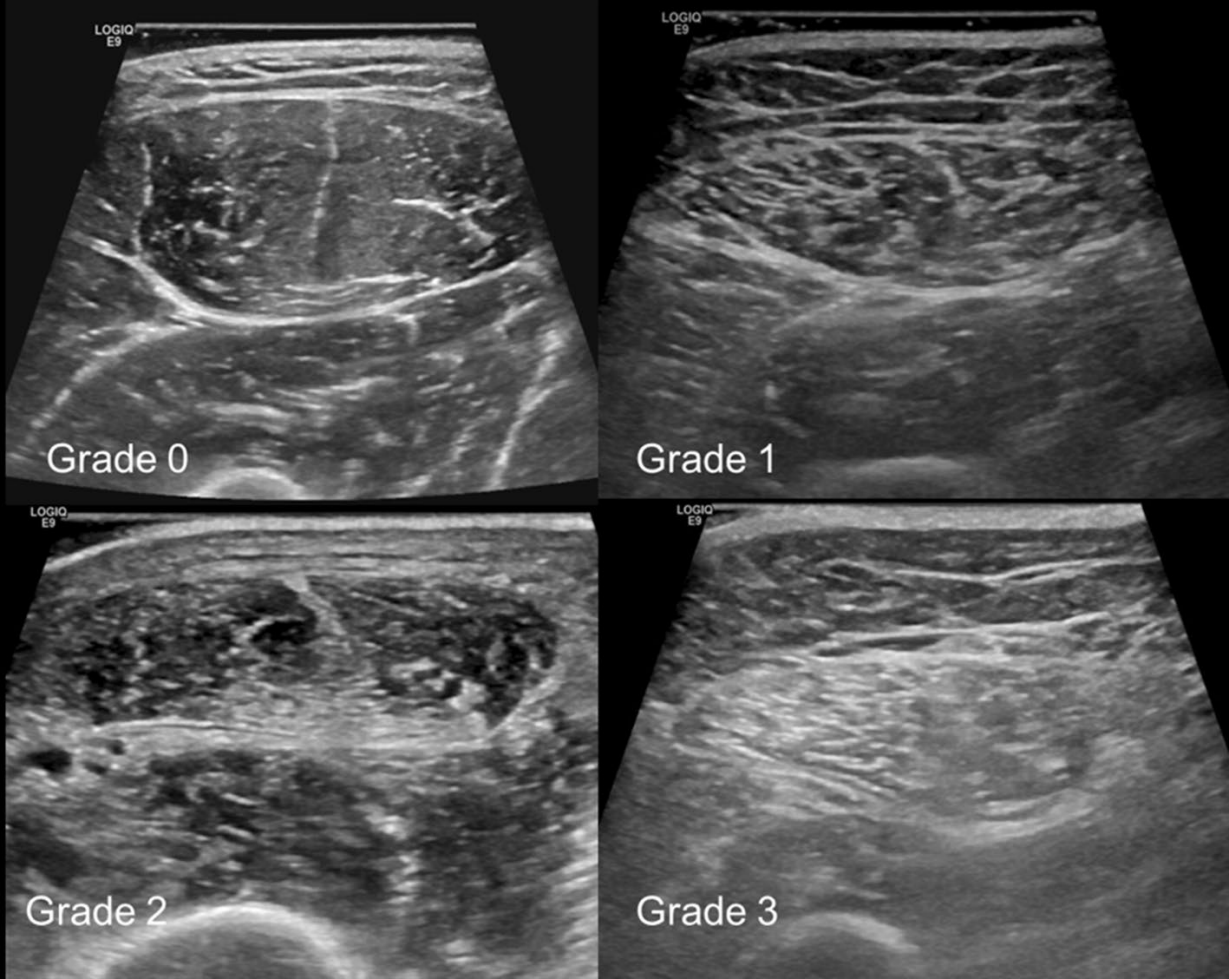
Muscle echogenicity grading on gray-scale ultrasonography of right mid rectus femoris muscle.

All subjects underwent USG evaluation at the mid-portion of the right rectus femoris muscle with the same protocol and US scanner (LOGIQ E9; GE Healthcare, Wauwatosa, WI, USA) using a linear 9- to 5-MHz probe. This was performed by a musculoskeletal radiologist with 5 years of experience. All subjects were examined in the supine position with a neutral foot position and were asked not to move or strain the lower extremity. The GSU and SWE images of the right mid rectus femoris muscle in a transverse plane were acquired simultaneously. Color box was placed on rectus femoris muscle and the visual color scale bar is in the left aspect of the screen (red means hard and blue means soft) (Fig. 4). During USG, copious gel was applied and without flattening or deforming the superficial epimysium layer to minimize the external pressure which might affect the measurement^27^. To obtain the ground truth muscle grade, two musculoskeletal radiologists (13 years and 5 years of musculoskeletal radiology experience) classified the muscle echogenicity using a four-grade scale by consensus on the GSU image: (1) grade 0: low echoic muscle with inner speckled appearance of the perimysial connective tissue, (2) grade 1: area of increased echo relative to background muscle, (3) grade 2: nearly as echogenic as perimysial fat, and (4) grade 3: isoechoic to fat (Fig. 5)^28^. The muscle echogenicity grades were dichotomized as low grade (grade 0 and grade 1) and high grade (grade 2 and grade 3). This system was used as the muscle echogenicity grading ground truth system.

#### Assessment of sarcopenia: muscle quantification on computed tomography

All examinations were performed in a center using two multidetector-row computed tomography on an axial plane including the L3 level: a 128-slice system (Definition AS+, Siemens Healthineers, Forchheim, Germany) and a 64-slice system (Discovery CT 750 HD, GE Healthcare, Waukesha, WI, USA). The scan parameters were as follows: fixed tube potential = 120 kVp; beam collimation = 128 × 0.6 mm and 64 × 0.625 mm, respectively; slice thickening of 5 mm without interval. To obtain the ground truth for sarcopenia diagnosis, the inferior end plate level of the L3 image was evaluated to measure the total abdominal muscle area on the axial image. The Asan-J software, developed based on ImageJ (NIH, Bethesda, MD, USA), was used to measure the skeletal muscle mass on the CT image (available at http://datasharing.aim-aicro.com/morphometry). Sarcopenia was defined using the L3 skeletal muscle index (cm^2^/m^2^), which is based on the Korean National Health and Nutrition Examination Study (KNHANES): $\le$ 49 cm^2^/m^2^ for men and $\le$ 31 cm^2^/m^2^ for women^29^.

### Pre-processing

**Figure 6:**
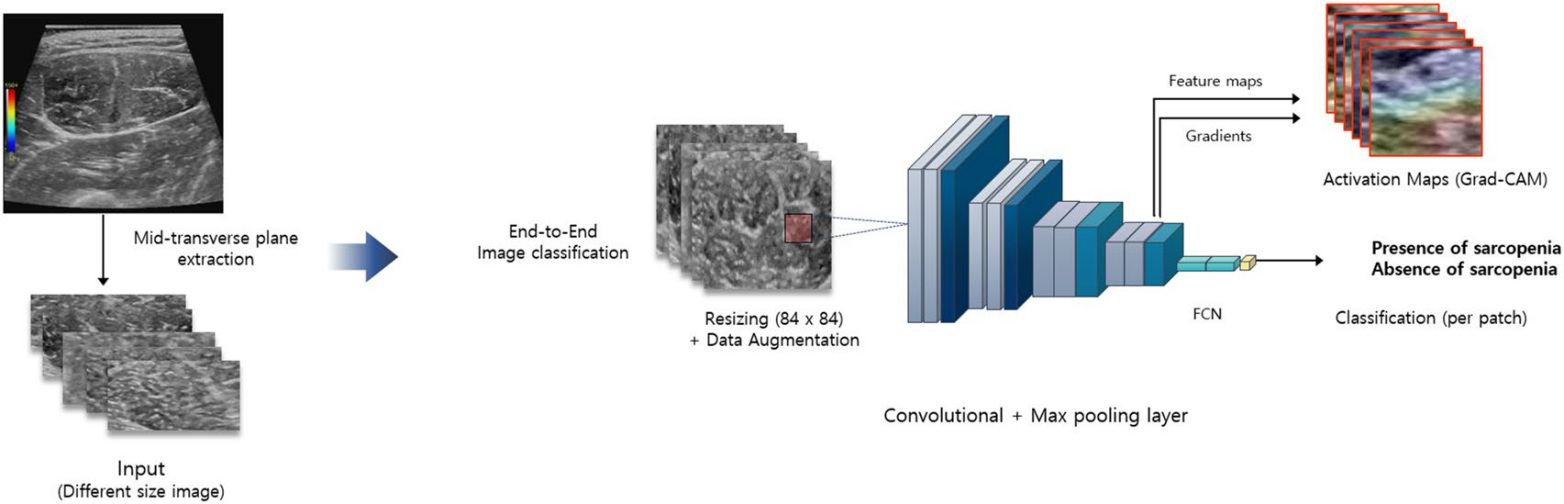
Schematic representation of the image pre-processing steps and deep neural network model for sarcopenia prediction.

Preprocessing techniques were applied for preparing the images to DCNN submission: (1) intensity normalization was performed in a nonlinear way to convert GSU and SWE images into standardized intensity ranges for all subjects, (2) image crop was applied for region-of-interest : elastographic areas in SWE images and ultrasonographic area in GSU images (except for text labels) (2) The dataset was augmented by applying a rotation (− 5° to 5°), shifting (shift limit of 0.0625), scaling (scale limit of 0.1), horizontal/vertical flipping, and additional random contrast, brightness, sharpness, blurring, and Gaussian noise to increase the generalization of our networks, (3) Finally, all images were resampled ass the retrieved ultrasonography images had different heights and widths. We resized the input images with 84 × 84 input size for SWE images and 112 × 112 input size for GSU images (Fig. 6). The pre-processing was performed using an in-house code written in MATLAB (Version 2018b, Math Works, Natick, MA, USA).

### DCNN architectures

Three different DCNNs (VGG19, ResNet-50, and DenseNet121) were used to classify sarcopenia muscle images to measure the efficacy of the DCNNs (Fig. 6). The pre-trained weights of all models were obtained from training a subset of ImageNet dataset—a large scale benchmark dataset that contains 1.28 million natural images with 1000 categories^30^. The saved DCNN models were then fine-tuned with the USG training dataset after replacing the final fully connected and softmax layer with a new fully connected layer and a random initialized sigmoid layer. The training for all the models was performed using a mini-batch gradient descent with Adam optimizer and a base learning rate of 0.0001 annealed by a factor of 0.1 when the gradient was stuck on a plateau. During the classification of the sarcopenia on GSU images, we utilized a gradient-weighted class activation map (Grad-CAM) technique to produce “visual explanations” for the decisions from our classification model^31^.

The pre-trained DCNN models were imported from the official repository in Keras, and all deep learning DCNN models were implemented using Keras (version.2.2.4) with a Tensorflow backend (version.1.10.0).

### Statistical analysis

Three DCNNs in each clinical situation were compared in accuracy. This was followed by a diagnostic performance evaluation of the DCNN architecture in sensitivity, specificity, positive and negative likelihood ratio, and area under the receiver operating characteristic curve (AUC). The best performing DCNN architecture for different clinical situations were selected based on accuracy and AUC. The considered clinical situations were (a) classification of normal vs. sarcopenia with GSU images; (b) classification of normal vs. sarcopenia with SWE images, and (c) muscle echogenicity grading (low vs. high) with GSU images. All statistical analyses were performed using R (version 3.6.2) (R Foundation for Statistical Computing, Vienna, Austria, https://www.R-project.org).

## Data Availability

The datasets generated during and/or analyzed during the current study are available from the corresponding author on reasonable request.
